# Association of Cardiovascular Risk Factors With Major and Minor Electrocardiographic Abnormalities: A Report From the Cross‐Sectional Phase of Tehran Cohort Study

**DOI:** 10.1002/hsr2.70350

**Published:** 2025-01-22

**Authors:** Pooria Ahmadi, Sajjad Ahmadi‐Renani, Parmida Sadat Pezeshki, Sepehr Nayebirad, Arash Jalali, Akbar Shafiee, Aryan Ayati, Arian Afzalian, Farshid Alaeddini, Soheil Saadat, Farzad Masoudkabir, Ali Vasheghani‐Farahani, Saeed Sadeghian, Mohamamdali Boroumand, Abbasali Karimi, Boshra Pourbashash, Kaveh Hosseini, Frits R. Rosendaal

**Affiliations:** ^1^ Department of Cardiology, Shariati Hospital, School of Medicine Tehran University of Medical Sciences Tehran Iran; ^2^ Tehran Heart Center, Cardiovascular Diseases Research Institute Tehran University of Medical Sciences Tehran Iran; ^3^ Department of Emergency Medicine University of California, Irvine Irvine California USA; ^4^ Cardiac Primary Prevention Research Center, Cardiovascular Diseases Research Institute Tehran University of Medical Sciences Tehran Iran; ^5^ Department of Clinical Epidemiology Leiden University Medical Center Leiden The Netherlands

**Keywords:** cardiovascular disease, diabetes, ECG, electrocardiogram, hypertension

## Abstract

**Background and Aims:**

In the current study, we aimed to identify the association between major and minor electrocardiographic abnormalities and cardiovascular risk factors.

**Methods:**

We used the Tehran cohort study baseline data, an ongoing multidisciplinary, longitudinal study designed to identify cardiovascular disease risk factors in the adult population of Tehran. The electrocardiograms (ECGs) of 7630 Iranian adults aged 35 years and above were analyzed. ECG abnormalities were categorized into major or minor groups based on their clinical importance. Results were obtained by multivariable logistic regression and are expressed as odds ratios (ORs).

**Results:**

A total of 756 (9.9%) participants had major ECG abnormalities, while minor abnormalities were detected in 2526 (33.1%). Males comprised 45.8% of the total population, and 41.8% of them had minor abnormalities. Individuals with older age, diabetes (OR = 1.35; 95% CI: 1.11–1.64), and hypertension (OR = 2.21; 95% CI: 1.82–2.68) had an increased risk of major ECG abnormalities. In contrast, intermediate (OR = 0.69; 95% CI: 0.57–0.84) and high physical activity levels (OR = 0.66; 95% CI: 0.51–0.86) were associated with a lower prevalence of major abnormalities. Male sex, older age, hypertension, and current smoking were also associated with an increased prevalence of ECG abnormalities combined (major or minor).

**Conclusion:**

Major and minor ECG abnormalities are linked with important cardiovascular risk factors such as diabetes and hypertension. Since these abnormalities have been associated with poor outcomes, screening patients with cardiovascular risk factors with an ECG may distinguish high‐risk individuals who require appropriate care and follow‐up.

## 1 Introduction

Cardiovascular diseases (CVDs) and coronary heart disease (CHD), in particular, are the primary contributors to increased disability‐adjusted life years (DALY) [1]. Major and minor electrocardiographic (ECG) abnormalities have been identified to be important predictors of CHD, cardiovascular mortality, stroke, and sudden cardiac death [2, 3, 4, 5, 6]. ECG abnormalities have been associated with an increased risk of CHD even after adjustment for other risk factors [2, 7, 8]. Due to worldwide availability, cheap cost, and ease of use, baseline ECG measurement can help identify high‐risk patients, especially those without symptoms [2, 3, 4, 5, 6]. For instance, in a nationwide Japanese study with a median follow‐up period of 5.5 years, abnormalities in the baseline ECG were found to be associated with overall mortality and hospital admissions because of CVD in the general population [9]. The diagnostic utility of ECG abnormalities for certain emerging CVDs has also been reported. Harms et al. [10] claimed that some ECG abnormalities in patients with type 2 diabetes without any pre‐existing CVD, could be associated with an emerging heart failure, while other abnormalities could precede CHD.

Minor and major ECG abnormalities have also been associated with modifiable and non‐modifiable CVD risk factors [11, 12, 13]; however, the literature surrounding the topic is scarce. Previous studies have demonstrated associations between modifiable risk factors such as high blood pressure and diabetes and major ECG abnormalities [11, 12, 13]. For instance, one study has shown that patients with uncontrolled or treatment‐resistant hypertension have a higher prevalence of major ECG abnormalities compared to normotensive people (prevalence ratio [PR] of 1.37 and 1.42, respectively). In the non‐modifiable CVD risk factors group, age has been the most prominent risk factor associated with major ECG abnormalities, but other risk factors such as male sex and black race may also increase the risk of ECG abnormalities [13, 14, 15, 16, 17]. Some CVD risk factors, such as older age, heart failure, or beta‐blocker use, have even been demonstrated to be predictors for a diagnostic Holter ECG monitoring [18].

Most studies on the association between ECG abnormalities and CVD risk factors have been conducted outside Middle Eastern countries. Since ethnic origin has been shown to affect ECG abnormalities [19, 20, 21], exploring these associations in different ethnicities is crucial. As a result, in the current study, we aimed to investigate the association between minor and major ECG abnormalities and CVD risk factors in the adult population of Tehran, the largest city in Iran. We used the data from the Tehran cohort study (TeCS), an ongoing cohort of Tehran's adult population specifically focused on CVDs and their risk factors.

## 2 Methods

### 2.1 Study Design and Participants

TeCS is a multidisciplinary, prospective, longitudinal cohort that started in 2016. A detailed version of the study methodology has been previously published [22, 23]. This was a cross‐sectional analysis of the recruitment phase of TeCS. The TeCS cohort was approved by the Tehran University of Medical Sciences ethical committee (Approval code: IR.TUMS.MEDICINE.REC.1399.074). In brief, 9548 households were randomly contacted from all regions of Tehran city by telephone number. A total of 4215 households agreed to enroll, from which 8296 individuals ≥ 35 years of age participated for cardiovascular and mental evaluation. The recruitment period of this study was from March 1, 2016, to March 1, 2019. All the participants provided written informed consent. For the current study, we excluded 666 participants with disqualified ECGs due to missing data, artifacts in the ECG, or lead reversals.

In a prior report, we provided the age and gender differences in individual ECG abnormalities from TeCS [24]. Major and minor abnormalities were more prevalent in males and older subjects; however, the categorization of abnormalities into major or minor groups differed from the current study. In addition, we showed that certain ECG parameters, such as P‐wave, QRS, and RR interval durations, were different between males and females. In the current analysis, we focused on the association of ECG abnormalities with CVD risk factors.

### 2.2 Data Collection and ECG Measurements

A 12‑lead ECG was obtained from the participants by a 12‐channel M‐TRACE ECG device (M4Medical, Lublin, Poland). Two expert cardiologists reviewed the saved electronic ECG documents on the TeCS server to investigate the ECG abnormalities for the current study. When the evaluations were inconsistent, they were reassessed by the third cardiologist.

Demographic and baseline characteristics of the participants were obtained from the TeCS database. Metabolic syndrome was defined based on the ATP iii criteria [25]. When the participant had three of the five following risk factors: elevated triglyceride, blood pressure, blood glucose, reduced high‐density lipoprotein, or high waist circumference, he was identified as having metabolic syndrome. Framingham risk score (FRS) was calculated based on the previous methodology, and participants were categorized into low (FRS < 10%), intermediate (10%–19%), and high (≥ 20%) risk groups [26]. Conventional CVD risk factors (hypertension, diabetes, hyperlipidemia, smoking, CHD family history) were also grouped as a variable named CVD risk factor number to assess the prevalence of ECG abnormalities in patients with different numbers of CVD risk factors. Coronary artery disease (CAD) was defined self‐reported, as previous coronary artery bypass surgery, percutaneous coronary intervention, myocardial infarction, or positive angiography report. Common medications with a potential impact on ECG parameters, including beta‐blockers, digoxin, and amiodaron use were also collected.

### 2.3 Definitions of the ECG Abnormalities

ECG interpretations were made according to the American Heart Association (AHA) recommendations [27]. ECG parameters (including RR intervals, heart rate [HR], QRS duration, and P‐wave duration) were assessed for each ECG document. Based on clinical significance, abnormal ECGs were classified into minor and major abnormality groups. Each ECG was evaluated for rhythm, axis, ST‐T abnormalities, and conduction problems. T wave abnormalities were grouped into ischemic T wave and other T wave inversions (secondary, strain, and nonspecific inversions). The major abnormality group included the ischemic T wave abnormalities (ST coving, biphasic T wave, symmetric T wave inversion, and giant T wave inversion). Furthermore, due to frequent nonspecific T wave inversions in AVR, V1, and III leads in healthy people, the ECG was considered with a minor or major abnormality if an inverted T wave was observed in 4 or ≥ 5 leads, respectively.

Patients with Q wave in 3 or ≥ 4 leads were included in the minor or major ECG abnormality group, respectively. Since the Q wave can be considered a normal variant in III and V1 leads, we decided to categorize the Q waves like this. Similarly, we included patients with ST depression in 2 or ≥ 3 leads in the minor or major ECG abnormality group, respectively. The ECG of the participants was evaluated in terms of the corrected QT interval (QTc) with the Bazzet formula. Patients with a QTc between 450 and 480 ms in women or 440 and 470 ms in men were included in the minor abnormalities group. In comparison, individuals with a QTc longer than 480 ms in women or 470 ms in men were incorporated into the major abnormalities group.

Patients were also included in the major ECG abnormality group under the category name (non‐sinus rhythm) if they had atrial fibrillation, atrial flutter, junctional rhythm, or pacemaker rhythm. Additionally, patients with complete heart block, high‐degree atrioventricular block (AVB), left bundle branch block (LBBB), right bundle branch block (RBBB), or intraventricular conduction delay were included in the major abnormality group. On the other hand, minor abnormalities included premature atrial contraction, premature ventricular contraction, incomplete RBBB, incomplete LBBB, abnormal QRS axis, first‐degree AVB, and Sinus bradycardia (HR < 60), left atrial enlargement, wandering pacemaker, or atrial rhythm. The complete categorization of the ECG abnormalities into major or minor groups is shown in Table 1. Patients with major and minor ECG abnormalities were only included in the major abnormalities group.

**Table 1:** Prevalence of major and minor electrocardiographic abnormalities in the study population.

	Number of participants (%)
Major abnormalities	
Major long QTc	50 (0.70%)
Non‐sinus rhythm	50 (0.66%)
High degree AVB	2 (0.03%)
Complete heart block	1 (0.01%)
IVCD	18 (0.24%)
T wave abnormalities	
Ischemic T wave	309 (4.05%)
Major T wave inversion	301 (3.94%)
RBBB	101 (1.32%)
LBBB	73 (0.96%)
Major Q wave	32 (0.42%)
Major ST depression	199 (2.61%)
Minor abnormalities	
First‐degree AVB	70 (0.92%)
Poor R wave progression	505 (6.62%)
LAE	360 (4.72%)
Incomplete LBBB	1 (0.01%)
Incomplete RBBB	218 (2.86%)
Wandering pacemaker	1 (0.01%)
PVC	95 (1.25%)
PAC	67 (0.88%)
Minor T wave inversion	299 (3.92%)
Minor ST depression	231 (3.03%)
Minor Q wave	109 (1.43%)
Minor long QT	500 (6.97%)
Atrial rhythm	24 (0.31%)
Sinus bradycardia	1145 (16.11%)
Abnormal QRS axis	464 (6.08%)

*Note:* Non‐sinus rhythm—atrial fibrillation, atrial flutter, junctional rhythm, or pacemaker rhythm; Ischemic T wave—ST coving, biphasic T wave, symmetric T wave inversion, giant T wave inversion; Major Q wave—Q wave ≥ 4 leads; Minor Q wave—Q wave in 3 leads; Major T wave inversion ≥ 5 leads; Minor T wave inversion—T wave inversion in 4 leads; Major ST depression—ST depression ≥ 3 leads; Minor ST depression—ST depression in 2 lead.

Abbreviations: AVB, atrioventricular block; IVCD, intraventricular conduction delay; LAE, left atrial enlargement; LBBB, left bundle branch block; PAC, premature atrial complex; PVC, premature ventricular complex; QTc, corrected QT interval; RBBB, right bundle branch block.

### 2.4 Statistical Analysis

We reported normally distributed continuous variables as mean (standard deviation) and non‐normal variables as median (interquartile range). Normality was assessed using central tendency measures as well as histogram charts. Normally distributed continuous variables were analyzed using a one‐way analysis of variance, followed by Dunnett's pairwise comparison between minor and major categories and the normal group. For skewed variables such as triglyceride and fasting blood sugar, the Kruskal–Wallis test was employed, followed by multiple median comparisons. Categorical variables were compared using chi‐square tests to assess differences between the minor, major, and normal groups. Fisher's exact test was applied specifically for the Amiodarone variable. Clinically important determinants of ECG abnormalities were entered in multivariable logistic regression analysis to adjust for possible confounders. All statistical tests were two‐sided with a priori significancy level of 0.05. Odds ratios (ORs) and their respective 95% confidence intervals (CIs) were then reported. We carried out the analysis with the IBM SPSS Statistics for Windows, version 23 (Armonk, NY: IBM Corp.).

## 3 Results

A total of 7630 patients were included in the final analyses, of whom 756 and 2526 had major and minor ECG abnormalities, respectively. The average age of the study participants was 53.59 ± 12.66, with females accounting for 4132 (54.15%) of the population. The most prevalent major abnormalities in our study group were ischemic T wave, major T wave inversion, and major ST depression observed in 4.05%, 3.94%, and 2.61% of participants, respectively. Similarly, sinus bradycardia was the most prevalent minor abnormality detected in 16.11% of participants, followed by poor R wave progression (6.62%), minor long QT interval (6.97%), and abnormal QRS axis (6.08%) being the next prevalent abnormalities (Table 1).

Table 2 demonstrates the prevalence of cardiovascular risk factors and comorbidities in the study population based on the presence of abnormalities in their ECG. Participants in the major ECG abnormality group were older than those in the normal and minor abnormality ECG groups. Men comprised 1704 (39.19%), 1462 (57.88%), and 332 (43.92%) of the normal, minor, and major ECG groups, respectively. The prevalence of hypertension, diabetes mellitus (DM), hyperlipidemia, and metabolic syndrome was highest in participants with major ECG abnormalities. High FRS was also more common among individuals with major ECG abnormalities 309 (43.28%) compared with minor and normal ECG groups (23.04% and 13.38%, respectively). In addition, a considerably higher number of patients in the major ECG abnormality group had 2 or > 2 CVD risk factors compared to the normal and minor abnormality group (Table 2).

**Table 2:** Demographic, clinical, and laboratory characteristics of the patients based on ECG abnormalities.

	Total (*N* = 7630)	Normal ECG (*N* = 4348)	Major abnormalities (*N* = 756)	Minor abnormalities (*N* = 2526)	*p*‐value	Minor versus Normal	Major versus Normal
Gender (male)	3498 (45.85%)	1704 (39.19%)	332 (43.92%)	1462 (57.88%)	**< 0.001**	**< 0.001**	**0.014**
Age	53.59 (12.66)	50.93 (11.54)	62.94 (13.36)	55.36 (12.66)	**< 0.001**	**< 0.001**	**< 0.001**
35–44	2173 (28.48%)	1519 (34.94%)	79 (10.45%)	575 (22.76%)	**< 0.001**	**< 0.001**	**< 0.001**
45–54	2053 (26.91%)	1260 (28.98%)	124 (16.4%)	669 (26.48%)			
55–64	1796 (23.54%)	960 (22.08%)	181 (23.94%)	655 (25.93%)			
> 65	1608 (21.07%)	609 (14.01%)	372 (49.21%)	627 (24.82%)			
HTN	2099 (27.57%)	940 (21.67%)	423 (56.03%)	736 (29.19%)	**< 0.001**	**< 0.001**	**< 0.001**
DM	1347 (17.97%)	680 (15.88%)	245 (33.15%)	422 (17.06%)	**< 0.001**	0.209	**< 0.001**
HLP	2456 (32.26%)	1257 (28.98%)	359 (47.61%)	840 (33.32%)	**< 0.001**	**< 0.001**	**< 0.001**
Metabolic syndrome	2116 (28.7%)	1129 (26.75%)	325 (45.58%)	662 (27.14%)	**< 0.001**	0.726	**< 0.001**
CAD FH	713 (9.34%)	420 (9.66%)	75 (9.92%)	218 (8.63%)	0.31	0.156	0.823
BMI under 25	2130 (28.19%)	1196 (27.7%)	208 (28.3%)	726 (28.98%)	**0.05**	0.299	**0.015**
Overweight	3173 (41.99%)	1861 (43.11%)	279 (37.96%)	1033 (41.24%)			
Obese and more	2254 (29.83%)	1260 (29.19%)	248 (33.74%)	746 (29.78%)			
Current smoker	985 (12.99%)	474 (10.97%)	93 (12.38%)	418 (16.67%)	**< 0.001**	**< 0.001**	0.256
FRS low	4446 (61.3%)	2896 (69.82%)	237 (33.19%)	1313 (54.91%)	**< 0.001**	**< 0.001**	**< 0.001**
Intermediate	1392 (19.19%)	697 (16.8%)	168 (23.53%)	527 (22.04%)			
High	1415 (19.51%)	555 (13.38%)	309 (43.28%)	551 (23.04%)			
CVD RF number (0)	2451 (33.18%)	1602 (37.88%)	117 (16.34%)	732 (29.96%)	**< 0.001**	**< 0.001**	**< 0.001**
1	2323 (31.44%)	1347 (31.85%)	180 (25.14%)	796 (32.58%)			
2	1494 (20.22%)	757 (17.9%)	203 (28.35%)	534 (21.86%)			
2<	1120 (15.16%)	523 (12.37%)	216 (30.17%)	381 (15.6%)			
Low PA	1309 (17.32%)	652 (15.13%)	238 (31.73%)	419 (16.75%)	**< 0.001**	**0.001**	**< 0.001**
Intermediate PA	4415 (58.41%)	2623 (60.89%)	384 (51.2%)	1408 (56.3%)			
High PA	1835 (24.28%)	1033 (23.98%)	128 (17.07%)	674 (26.95%)			
Systolic BP	121.71 ± 18.88	119.34 ± 17.77	130.05 ± 21.47	123.31 ± 19.04	**< 0.001**	**< 0.001**	**< 0.001**
Diastolic BP	80.76 ± 10.81	80.59 ± 10.29	82.94 ± 12.6	80.41 ± 11.04	**< 0.001**	0.764	**< 0.001**
LDL	113.38 ± 34.08	114.33 ± 33.22	108.64 ± 36.73	113.15 ± 34.63	**< 0.001**	0.315	**< 0.001**
HDL	44.83 ± 12.37	45.2 ± 12.29	44.48 ± 12.9	44.31 ± 12.34	**0.01**	**0.010**	0.274
TG	124 (88–174)	124 (87–173)	130 (91–184)	123 (88–174)	**0.04**	**0.778**	**0.037**
Total cholesterol	172.51 ± 40.35	173.4 ± 39.23	169.06 ± 43.52	171.99 ± 41.24	0.22	0.310	**0.015**
FBS	97 (90–107)	96 (90–105)	101 (92–122)	97 (91–107)	**< 0.001**	0.071	**< 0.001**
BMI	27.95 ± 4.81	27.96 ± 4.72	28.18 ± 5.05	27.86 ± 4.9	0.28	0.535	0.430
Waist‐to‐hip ratio	0.91 ± 0.07	0.91 ± 0.07	0.93 ± 0.07	0.92 ± 0.07	**< 0.001**	**< 0.001**	**< 0.001**
Waist circumference	96.13 ± 11.72	95.5 ± 11.61	97.47 ± 12.13	96.83 ± 11.74	**< 0.001**	**< 0.001**	**< 0.001**
Beta‐blocker use (yes)	1258 (16.5%)	547 (12.6%)	244 (32.3%)	467 (18.5%)	**< 0.001**	**< 0.001**	**< 0.001**
Amiodaron	18 (0.2%)	6 (0.1%)	6 (0.8%)	6 (0.2%)	**0.003**	0.376	**0.004**
Digoxin	49 (0.6%)	6 (0.1%)	32 (4.2%)	11 (0.4%)	**< 0.001**	**0.017**	**< 0.001**
CAD	574 (7.5%)	179 (4.1%)	185 (24.5%)	210 (8.3%)	**< 0.001**	**< 0.001**	**< 0.001**

*Note:* Bold values represent *p*‐values < 0.05.

Abbreviations: BMI, body mass index; BP, blood pressure; CAD, coronary artery disease; CAD FH, family history of coronary artery disease; CVD RF, cardiovascular risk factors; DM, diabetes mellitus; FBS, fasting blood sugar; FRS, Framingham risk score; HDL, high‐density lipoprotein; HLP, hyperlipidemia; HTN, hypertension; LDL, low‐density lipoprotein; PA, physical activity.

Logistic analysis showed that higher age groups, DM (OR = 1.27; 95% CI: 1.04–1.55) and hypertension (OR = 1.84; 95% CI: 1.50–2.27), history of CAD (OR = 2.62; 95% CI: 2.04–3.35), and digoxin use (OR = 9.71; 95% CI: 4.76–19.80) were associated with an increased prevalence of major ECG abnormalities. Intermediate (OR = 0.73; 95% CI: 0.59–0.89) and high physical activity levels (OR = 0.73; 95% CI: 0.56–0.94) were associated with a lower prevalence of major abnormalities (Table 3). Male sex, older age, hypertension, current smoking, history of CAD, beta‐blocker use, and digoxin use were associated with a higher prevalence of ECG abnormalities combined (Table 4).

**Table 3:** Multivariable logistic regression analysis of clinically important variables on major ECG abnormalities.

	Odds ratio	95% Confidence interval	*p*‐value
Gender (male)	0.76	0.63–0.91	**0.004**
Age 35–44	Reference		**< 0.001**
45–54	1.40	1.03–1.90	**0.03**
55–64	1.87	1.38–2.53	**< 0.001**
> 65	3.92	2.91–5.27	**< 0.001**
DM	1.27	1.04–1.55	**0.02**
HTN	1.84	1.50–2.27	**< 0.001**
HLP	0.94	0.78–1.14	0.56
BMI under 25	Reference		0.18
Overweight	0.83	0.67–1.03	0.09
Obese and more	0.82	0.65–1.04	0.10
Waist‐to‐hip ratio	1.07	0.85–1.35	0.55
Current smoker	1.09	0.83–1.42	0.53
Low PA	Reference		**0.007**
Intermediate PA	0.73	0.59–0.89	**0.002**
High PA	0.73	0.56–0.94	**0.02**
CAD FH	1.14	0.86–1.50	0.37
CAD	2.62	2.04–3.35	**< 0.001**
Beta‐blocker use	1.06	0.86–1.31	0.61
Amiodaron	1.29	0.36–4.64	0.70
Digoxin	9.71	4.76–19.80	**< 0.001**

*Note:* The Hosmer and Lemeshow test was performed to evaluate the logistic regression model's goodness of fit with a chi‐square value of 9.60 with 8 degrees of freedom (*p* = 0.30). Bold values represent *p*‐values < 0.05.

Abbreviations: BMI, body mass index; CAD FH, family history of coronary artery disease; DM, diabetes mellitus; HLP, hyperlipidemia; HTN, hypertension; PA, physical activity.

**Table 4:** Multivariable logistic regression analysis of clinically important variables on ECG abnormalities (major or minor).

	Odds ratio	95% Confidence interval	*p*‐value
Gender (male)	1.82	1.63–2.02	**< 0.001**
Age 35–44	Reference		**< 0.001**
45–54	1.35	1.18–1.54	**< 0.001**
55–64	1.74	1.51–2.01	**< 0.001**
> 65	2.87	2.44–3.37	**< 0.001**
DM	0.89	0.78–1.03	0.11
HTN	1.28	1.12–1.46	**< 0.001**
HLP	1.05	0.94–1.18	0.38
BMI under 25	Reference		0.10
Overweight	0.91	0.8–1.03	0.13
Obese and more	1.03	0.9–1.18	0.70
Waist‐to‐hip ratio	0.93	0.82–1.05	0.26
Current smoker	1.20	1.03–1.4	**0.02**
Low PA	Reference		**0.01**
Intermediate PA	0.86	0.75–0.99	**0.03**
High PA	1.00	0.86–1.18	0.96
CAD FH	1.02	0.86–1.21	0.83
CAD	1.57	1.26–1.94	**< 0.001**
Beta‐blocker use	1.27	1.09–1.48	**0.002**
Amiodaron	0.87	0.28–2.71	0.81
Digoxin	6.28	2.41–16.37	**< 0.001**

*Note:* The Hosmer and Lemeshow test was performed to evaluate the logistic regression model's goodness of fit with a chi‐square value of 4.26 with 8 degrees of freedom (*p* = 0.83). Bold values represent *p*‐values < 0.05.

Abbreviations: BMI, body mass index; CAD FH, family history of coronary artery disease; DM, diabetes mellitus; HLP, hyperlipidemia; HTN, hypertension; PA, physical activity.

## 4 Discussion

**Figure 1:**
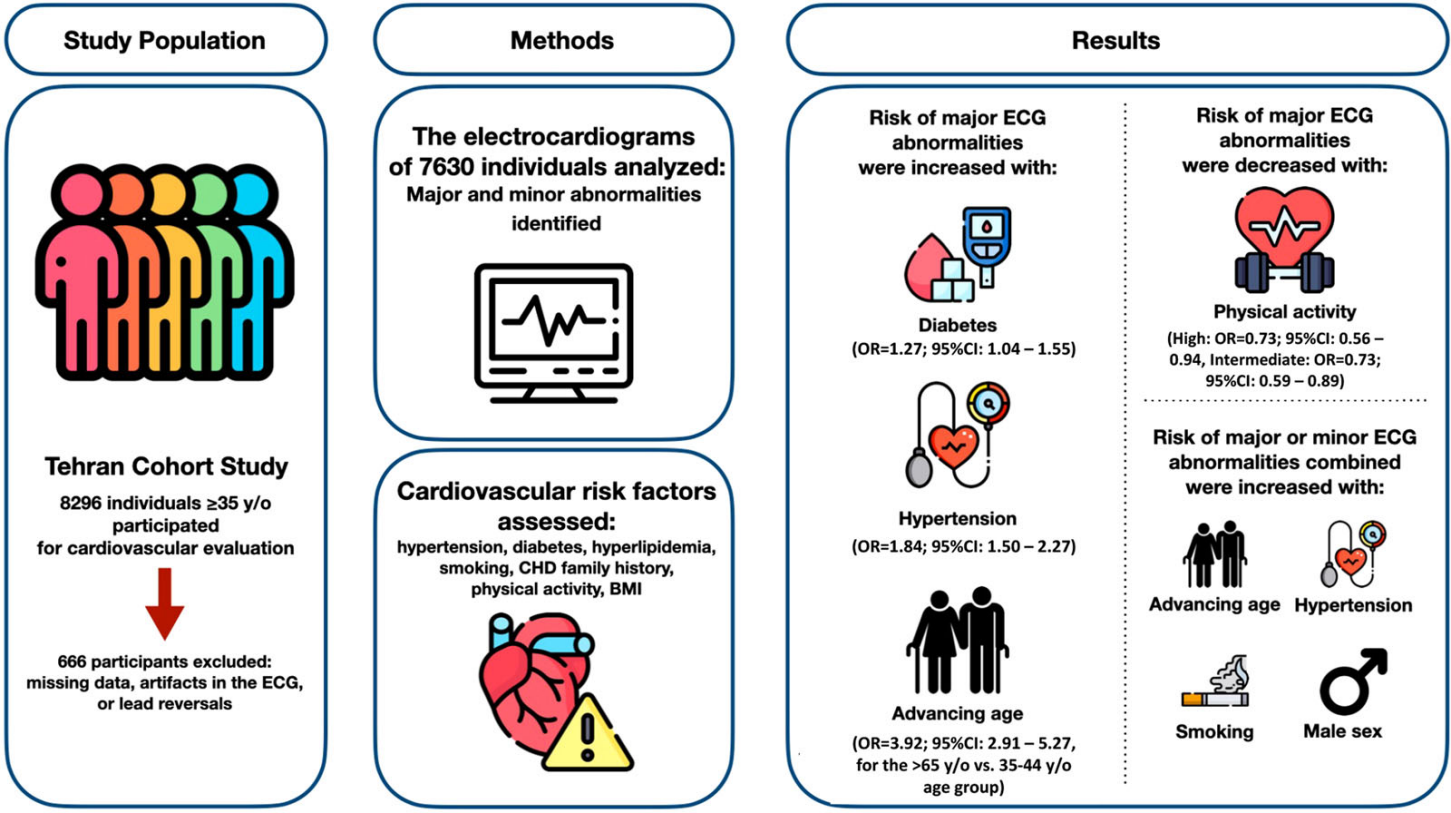
The graphical abstract, summarizing the design and main results of our study.

Identifying individuals at risk of major ECG abnormalities is relevant as these abnormalities predict mortality risk in various patient settings [28, 29, 30, 31]. In the present study, we investigated the association between ECG abnormalities and CVD risk factors using data from the TeCS cohort, a random sample of Tehran's population. Our study demonstrated a higher prevalence of ECG abnormalities in individuals with cardiovascular risk factors, including older age, hypertension, DM, dyslipidemia, metabolic syndrome, current smoking, and low physical activity, than in individuals without these risk factors. In addition, we demonstrated that the prevalence of ECG abnormalities was higher in individuals with more CVD risk factors or higher FRS than in those without. Figure 1 summarizes the design and main results of the study.

After adjustment for possible confounders, male sex, older age, hypertension, history of CAD, beta‐blocker use, digoxin use, and low physical activity were associated with major and minor ECG abnormalities combined. For major abnormalities alone, older age, diabetes, hypertension, and low physical activity were identified as predictors. As expected, major and minor abnormalities groups had a lower prevalence of females than the normal ECG group (56% and 42% vs. 61%, respectively) [32].

The prevalence of major ECG abnormalities was almost two times higher in hypertensive patients than in normotensives (OR = 1.84; 95% CI: 1.50–2.27). This finding was in line with the results of the previous studies [2, 5, 11, 33, 34]. Yu et al. [33] showed that major and minor arrhythmias were 1.29 times more prevalent in hypertensive individuals than in the normotensive population. Furthermore, this study demonstrated that hypertension was associated with other ECG abnormalities such as ST depression and T abnormalities, Q wave abnormalities, and tall R wave [33]. Other studies have proposed that hypertension may be the modifiable CVD risk factor most associated with ECG abnormalities, particularly nonspecific ST segment and T wave abnormalities [2, 5, 35]. The findings by Bhatt et al. [11] suggested that the prevalence of ECG abnormalities was associated with hypertension severity and blood pressure control. While the prevalence of major ECG abnormalities was not different between prehypertension and normotension groups, the prevalence in controlled and uncontrolled hypertension groups was higher (PR = 1.62 and 1.64, respectively) than in normotensive individuals [11]. In the apparent treatment‐resistant hypertension group, this figure rose to 2.12 [11].

It has been suggested that uncontrolled hypertension can lead to increased left atrial stretch, size, and left ventricular hypertrophy, resulting in electrical and structural remodeling of myocardiocytes [36, 37]. The remodeling can then cause a variety of arrhythmias, such as atrial fibrillation or other supraventricular and ventricular arrhythmias [36]. Our study's major ECG abnormalities group included both arrhythmic and ischemic ECG indicators, and these abnormalities were strongly associated with hypertension independent of other risk factors.

Individuals with diabetes had higher odds of major ECG abnormalities than non‐diabetics, with an OR of 1.27. Studies have demonstrated that minor and major ECG abnormalities are prevalent in diabetic patients. In the Hoorn Diabetes Care System cohort, 16.0% and 13.1% of diabetic patients had minor and major ECG abnormalities, respectively [38]. A study on Angolan people identified the prevalence of minor and major ECG abnormalities in the diabetic subpopulation at 9.3% and 17.4% [35]. Other studies on diabetic patients have estimated the prevalence of minor and major abnormalities ranging from 12.5% to 37.0% and from 7.5% to 23%, respectively [39, 40, 41]. In the current study, the prevalence of minor abnormalities in diabetic patients was 31.3%, while the figure for major abnormalities was 18.2%. The disparities in the reported numbers can be attributed to the various categorizations of major and minor ECG abnormalities in different studies. Although the role of diabetes in ischemic heart disease is well‐known, its effects on the electrophysiologic characteristics of the heart are less understood [42]. It has been suggested that increased glucose levels can cause structural remodeling and mitochondrial dysfunction, resulting in slowed conduction velocity in diabetic patients [42]. This might also explain the variation in the prevalence of ECG abnormalities, as the level of glycemic control might differ among the studies.

Besides diabetes and hypertension, low physical activity was associated with major ECG abnormalities. The intermediate and high physical activity levels were protective against major ECG abnormalities, with ORs of 0.73 and 0.73, respectively. Few studies have assessed the effects of physical activity on ECG abnormalities in the normal population. The study in the Angolan population reported no associations between physical activity and ECG abnormalities [35]. Another study in Brazil also reported no associations [43]. In some populations, such as Iranians, patients with ECG abnormalities who are likely to be diagnosed with CVDs may be culturally discouraged from physical activity because of their heart condition. Thus, low physical activity may result from ECG abnormality and not vice versa. It is also likely that by lowering the burden of other cardiovascular risk factors, such as hypertension or diabetes, physical activity can protect against developing ECG abnormalities. Further prospective studies are required to confirm this hypothesis.

Our study also found a significant association between a history of CAD and the presence of major ECG abnormalities. This aligns with existing literature, which has consistently shown that ECG abnormalities like ischemic T‐wave inversions, ST‐segment depression, and Q waves are prevalent in CAD patients and can also act as key predictors for future cardiac events [2, 44]. For instance, Kaolawanich et al. [45] have shown that ECG abnormalities can be strong indicators of myocardial infarction and major adverse cardiac events in patients with known or suspected CAD. Our findings add to this body of knowledge by emphasizing that a history of CAD significantly elevates the risk of detecting major and combined ECG abnormalities in a diverse adult population. This underscores the value of incorporating CAD history into ECG interpretation for better risk stratification and patient management.

In terms of medication use, digoxin stood out as having a strong association with major ECG abnormalities. This finding is consistent with previous research that has identified digoxin as a common cause of ECG changes, including down‐sloping ST‐T segment abnormalities and disturbances in both atrioventricular and intraventricular conduction, due to its impact on myocardial repolarization and arrhythmogenesis [46]. On the other hand, beta‐blocker use was linked to a moderate increase in ECG abnormalities when considering both major and minor findings, although it did not reach statistical significance for major abnormalities alone. This was expected as beta‐blockers are expected to cause sinus bradycardia and PR‐interval prolongation (both minor ECG abnormalities) rather than any major changes. This could reflect the role of beta‐blockers in clinical practice, where they are used for managing arrhythmias, and demonstrates that they can also lead to ECG changes that are not necessarily clinically significant. Amiodarone, despite its known effects on cardiac electrophysiology, did not show a significant correlation with ECG abnormalities in our analysis, potentially due to a limited number of only 18 cases who took the drug in our study.

Several studies have suggested that obesity is associated with various ECG abnormalities, particularly low QRS voltage, left ventricular hypertrophy, long QT interval, changes in P wave morphology, and leftward axis shift of all three major ECG waves (P, QRS, and T waves) [47]. However, recent evidence suggests obesity alone may not be as important as initially thought, and metabolic health plays a more significant role [48]. In the current study, obesity was not associated with a higher prevalence of ECG abnormalities, which may be due to the different analyzed ECG parameters. In addition, we did not assess the impact of metabolic health on ECG abnormalities in obese individuals.

### 4.1 Limitations

The cross‐sectional analysis of the current study was a major limitation, as no temporal associations could be established between ECG abnormalities and CVD risk factors, and reverse causation could have played a role in some associations, for example, with physical exercise. Second, the study population included Tehran adult residents > 35 years old, and the results can not be generalized to younger adults and the total Iranian population. Third, ECG interpretation was not automated, but it was undertaken by two expert cardiologists. Fourth, the categorization of ECG abnormalities into major or minor subgroups was based on the clinical relevance of each abnormality and not previously established criteria. Finally, we did not have complete data on variables such as history of heart failure, other antiarrhythmic medications, and history of ablation, which may have affected the ECG.

## 5 Conclusion

Major and minor ECG abnormalities were linked with important cardiovascular risk factors such as DM and hypertension. Since these abnormalities have been associated with poor outcomes, screening patients with cardiovascular risk factors using ECG can help distinguish high‐risk individuals and provide appropriate care and follow‐up.

## Author Contributions

Pooria Ahmadi, Sepehr Nayebirad, Parmida Sadat Pezeshki, and Aryan Ayati contributed to writing–original draft. Akbar Shafiee, Arian Afzalian, Kaveh Hosseini, and Frits R. Rosendaal contributed to writing–review and editing. Parmida Sadat Pezeshki contributed to the visualization. Pooria Ahmadi, Sajjad Ahmadi‐Renani, Ali Vasheghani‐Farahani, Kaveh Hosseini, and Boshra Pourbashash contributed to data curation and methodology. Kaveh Hosseini contributed to the conceptualization. Akbar Shafiee, Soheil Saadat, Saeed Sadeghian, Farshid Alaeddini, and Mohamamdali Boroumand contributed to the project administration and validation. Arash Jalali contributed to the formal analysis. Farzad Masoudkabir and Abbasali Karimi contributed to the supervision. All authors completely reviewed the manuscript and approved the version to be published.

## Ethics Statement

The TeCS cohort was approved by the Tehran University of Medical Sciences ethical committee with the following code: IR.TUMS.MEDICINE.REC.1399.074.

## Consent

Written informed consent was obtained from every participant.

## Conflicts of Interest

The authors declare no conflicts of interest.

## Data Availability

The data set of the present study is only available upon reasonable request from the corresponding author due to the current policy of the Tehran Heart Center.
